# Utilizing T_1_- and T_2_-Specific Contrast Agents as “Two Colors” MRI Correlation

**DOI:** 10.3390/ma18102290

**Published:** 2025-05-14

**Authors:** Adriaan L. Frencken, Barbara Blasiak, Boguslaw Tomanek, Danuta Kruk, Frank C. J. M. van Veggel

**Affiliations:** 1 Department of Chemistry, University of Victoria, Victoria, BC V8W 2Y2, Canada; 2 Centre for Advanced Materials & Related Technologies (CAMTEC), University of Victoria, Victoria, BC V8W 2Y2, Canada; 3 Experimental Imaging Centre, University of Calgary, Calgary, AB T2N 4N1, Canada; tomanek@ualberta.ca; 4 Institute of Nuclear Physics, Polish Academy of Sciences, 31-342 Krakow, Poland; 5 Department of Oncology, University of Alberta, Edmonton, AB T6G 2T4, Canada; 6 Department of Physics and Biophysics, University of Warmia and Mazury in Olsztyn, Oczapowskiego 4, 10-719 Olsztyn, Poland; danuta.kruk@uwm.edu.pl

**Keywords:** MRI, contrast agents, MRI correlation, relaxation times

## Abstract

Magnetic resonance imaging (MRI) is widely used as a medical imaging technique due to its non-invasive nature, high spatial contrast, and virtually unlimited depth of penetration. Different modalities can be used for contrast in MRI, including *T*_1_ (spin–lattice) and *T*_2_ or *T*_2_ * (spin–spin) proton relaxation times, and specific contrast agents (CAs) have been developed that locally enhance the contrasts in MRI images. We present a method combining *T*_1_- and *T*_2_-specific CAs in a single imaging technique, referred to as correlation MRI. This technique allows different CAs to be used simultaneously to visualize contrast between multiple types of tissue in the same image when applied as targeted CAs. An obstacle for the quantitative use of correlation MRI is that *T*_1_ and *T*_2_ relaxivity changes generated by CAs are not independent of each other. Here, we measured relaxivities in mixtures with various concentrations of Cas, including Magnevist (Gd^3+^-based, primarily a T_1_ CA) and Feridex (Fe^2+^- and Fe^3+^-based, primarily a T_2_ CA), and compared them to theoretically predicted values. It was found that, at clinically relevant concentrations, relaxivities of the mixtures deviate from linearly added values. We finally propose a three-dimensional calibration curve to quantitatively determine the concentration in mixtures of CAs, based on the measured relaxivities.

## 1. Introduction

Since its first demonstration in 1977, magnetic resonance imaging (MRI) has seen widespread use as a medical imaging technique [1,2]. In MRI, images are obtained by spatially encoding proton spins in a gradient of the external magnetic field [2]. The advantages of MRI are a high soft tissue contrast and unlimited depth of tissue penetration, all while being non-invasive to the body. A disadvantage of MRI compared with other medical imaging techniques is its low sensitivity. Increasing the sensitivity of MRI has been an outstanding challenge in the field. Strategies to increase the sensitivity include increasing the strength of the applied magnetic field [3,4] and utilizing chemical contrast agents (CAs) that locally enhance the MRI contrast [5,6,7,8].

*T*_1_, *T*_2_, and *T*_2_ * relaxations are common parameters to generate MRI contrast. In *T*_1_-weighted MRI, contrast is derived from the relaxation of a water proton spin with respect to the external magnetic field, referred to as spin–lattice relaxation time. *T*_2_ and *T*_2_ * are derived from relaxation times of water proton spins with respect to the surrounding water proton spins, also referred to as spin–spin relaxation in the absence (*T*_2_) and presence (*T*_2_ *) of local field inhomogeneities. Hence, *T*_2_ * includes the contribution which is unrelated to tissue [9].

Over the years, CAs enhancing *T*_1_ [10,11] or the *T*_2_ [12,13] relaxation of water protons have been developed for T_1_- and T_2_-weighted MRI. Using these CAs, *T*_1_- and *T*_2_-weighted images can be generated that accurately map the presence of such a CA, showing contrast against surrounding tissue.

The contrast generated by individual CAs is described in Equations (1) and (2). Here, the overall relaxation rate (i.e., 1/*T*_1_ or 1/*T*_2_) depends on the specific relaxivity of the contrast agent *r*_1_ or *r*_2_, the concentration of the contrast agent [CA], and the background relaxation rates 1/*T*_1_(0) and 1/*T*_2_(0). Using these equations, individual CAs can be quantified based on MRI contrast.

(1) $$\frac{1}{T_{1}}=\frac{1}{T_{1}\left(0\right)}+r_{1}\times [CA]$$

(2) $$\frac{1}{T_{2}}=\frac{1}{T_{2}\left(0\right)}+r_{2}\times [CA]$$

The existence of these two properties in MRI and the ability to quantify CAs based on them has served as an inspiration to us and others to develop an MRI contrast correlation technique. Image correlation analysis is applied in a variety of fields and entails the analysis of contrast in multiple images to derive a relationship between two or more parameters. It has been used to combine different modalities in medical imaging techniques [14,15] and is used in fluorescence correlation microscopy [16,17]. In a similar way, image correlation analysis could potentially be applied to combine different parameters within MRI, leading to a way to simultaneously visualize both parameters as two distinct types of contrast. This application has recently started to be explored [18,19,20,21]. Ideally, correlation spectroscopy utilizes two different colors that are fully independent of each other, for instance a blue color and a red color with no spectral overlap. In the same fashion, *T*_1_ and *T*_2_ parameters could serve as the “two colors” in MRI correlation. Some MRI contrast agents predominantly affect *T*_1_ relaxation with a small impact on *T*_2_ and vice versa, but having a pure effect on either modality is theoretically impossible [22,23].

The imaging technique discussed here combines the imaging of high *T*_1_ and high *T*_2_ contrast agents into an MRI correlation image. The relationship between *T*_1_ and *T*_2_ contrast generated by the contrast agents in the respective *T*_1_- and *T*_2_-weighted images can be analyzed and their correlation imaged. Combining these imaging techniques may lead to the visualization of phenomena in the body that would not be demonstrable, with high fidelity, using strictly *T*_1_ or *T*_2_. For example, two different tissues may be labeled with *T*_1_ or *T*_2_ contrast agents and their interaction monitored. This way, a tumor and its surrounding tissue may both be labeled to increase the tumor’s definition, or the heterogeneous receptors at a tumor’s surface may be labeled with two different contrast agents to outline the entire tumor. Furthermore, the exchange of CAs can be measured. There is also the clinical interest in the vascularity of tumors, and using two contrast agents, a tumor can be outlined with one CA and the blood vessels inside with another CA. In addition to these applications, MRI correlation would be beneficial when used for the measurement of a property inside tissue, such as pH or a biomolecule concentration, where the tissue contrast due to the presence of the CA is associated with the measured property. Hence, correlation can be used to co-introduce a control CA, that is independent of the property being measured, to verify the concentration of CAs at the location of interest. In short, there are many reasons to develop MRI correlation for clinical use, although it is fair to state that we are just at the beginning of this development.

Previous work has been conducted on the MRI correlation concept by Anderson et al. [19]. In an in vivo demonstration, Gd-BOPTA (gadobenic acid) [24] and Dy-DOTA-azide (dysprosium dodecane tetraacetic acid azide) contrast agents were injected in a mouse glioma model. Using both *T*_1_- and *T*_2_-weighted MRI, the concentrations of the injected contrast agents in the tumor could be mapped with reasonable accuracy (standard deviations of 10–16% in 10 demonstrations). The authors assumed a model where the relaxivity contributions of the contrast agents could be added linearly to arrive at the final measured relaxivity, which they argue to be valid if the two contrast agents have statistically different relaxivity ratios (*r*_2_/*r*_1_). The *r*_2_/*r*_1_ values they used were 374 for Dy-DOTA-azide and 6.59 for Gd-BOPTA with no reported overlap in the uncertainties of the ratios used. The linear model used is given by Equations (3) and (4).

(3) $$\frac{1}{T_{1}}=\frac{1}{T_{1}\left(0\right)}+r_{1,1}\times \left[CA1\right]+r_{1,2}\times [CA2]$$

(4) $$\frac{1}{T_{2}}=\frac{1}{T_{2}\left(0\right)}+r_{2,1}\times \left[CA1\right]+r_{2,2}\times [CA2]$$

where $r_{1,1}$ and $r_{1,2}$ denote the spin–lattice relaxivity for CA1 and CA2, respectively, and analogously for the spin–spin relaxivity, $r_{2,1}$ and $r_{2,2}$.

The demonstration using linear addition is valid only if all variables are independent of each other. This assumption may not be generally applicable, as the *r*_1_ and *r*_2_ of a specific contrast agent solute are never fully independent of each other, as they are described using a similar set of physical parameters [22,23].

Furthermore, the interaction of CAs with each other may influence their respective relaxivities as well. It is also expected that deviations from linear behavior are exacerbated at higher magnetic field strengths, due to relatively stronger increases in *r*_2_ with respect to *r*_1_ [12,25], with the trend that r_1_ generally plateaus or decreases at lower magnetic field strengths than *r*_2_ [26]. Such deviations from linearity may contribute to the standard deviations the authors report, which are quite high for a reliable use of the MRI correlation technique.

In this work, we test the validity of this model that linearly adds *r*_1_ and *r*_2_ values to quantify two commercially available contrast agents based on the relaxivities of their mixtures. We built on the previous demonstration by proposing a calibration model to accurately determine concentrations of two CAs in MRI to arrive at a reliable MRI correlation technique. The calibration method aims to verify and correct for potential concentration ranges where the respective relaxivities are not linearly additive.

Here, *T*_1_ and *T*_2_ contrast in concentration ranges of the *T*_1_ CA Magnevist (based on a Gd^3+^ multidentate complex) and *T*_2_ CA Feridex (based on iron oxide nanoparticles) are measured, and relaxivities are determined. The relaxation times of the mixtures of the CAs are measured as well, and the validity of linearly added relaxivities was determined by comparison of the experiment with the model. Finally, a calibration curve of the two contrast agents is presented, and its use to determine concentrations from *T*_1_ and *T*_2_ combinations is demonstrated.

## 2. Materials and Methods 

### 2.1. Chemicals

Feridex I.V.^®^ (Bayer Healthcare Pharmaceuticals Inc., Leverkusen, Germany) (ferumoxides injectable solution) and Magnevist-MAGNEVIST^®^ (brand of gadopentetate dimeglumine) (Bayer Healthcare Pharmaceuticals Inc., Leverkusen, Germany) were used. Double distilled water was used for dilutions.

### 2.2. Relaxation Time Determination

T_1_ and T_2_ were measured from a series of Feridex and Magnevist samples using a preclinical 9.4 T MRI system based on a 21 cm magnet (Magnex, Oxford, UK) and a Bruker console (Bruker, Karlsruhe, Germany). The water solutions of NPs were placed in 5 mm NMR tubes. A transmit/receive radio frequency birdcage coil was used for proton excitation and to collect the MR signal. For the *T*_1_ measurement, a *T*_1_-FISP echo sequence was used with the following parameters: 1.5 ms echo time (TE), 3 ms repetition time (TR), 15° flip angle (FA), a 3.00 by 3.00 cm field of view (FOV), and in a 128 by 128 matrix size. *T*_2_ was measured using a *T*_2_-MSME sequence, with the following parameters: 5.7 ms echo time (TE), 5000 ms repetition time (TR), 15° flip angle (FA), a 3.00 by 3.00 cm field of view (FOV), and a 128 by 128 matrix size. The monoexponential fitting (Bruker, Germany) method was applied for the relaxation calculations.

### 2.3. Data Analysis and Fitting

Three-dimensional plots and contour color maps were generated with both Origin and Matlab. Linear functions to data of pure dilutions of Feridex and Magnevist were carried out in Origin (OriginPro 2021) using an unweighted linear model. Error bars were assigned in the plots based on the measurement error in the *T*_1_ and *T*_2_ determination. Polynomial functions were fitted to 3-dimensional experimental data using Matlab (Matlab R2020a). For the calibration curve, a second-degree polynomial was optimized with five fitting parameters in the polynomial equation; see Equation (S1).

## 3. Results and Discussion

### 3.1. Relaxivities of Pure CAs

The commercially available contrast agents Feridex and Magnevist were chosen for the correlation demonstration. Feridex consists of iron oxide nanoparticles stabilized with citrate in water. The iron oxide has a magnetite structure and the average chemical formula FeO_1.44_. The commercially available dispersion contains 11.2 mg/mL iron (0.2 M). Magnevist comprises a solution of gadopentate dimeglumine (Gd-DTPA) at a 0.5 M concentration in water.

**Figure 1:**
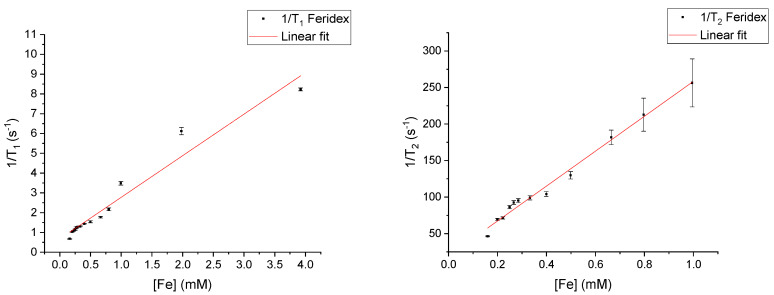
Relaxivity plots showing measured 1/*T*_1_ (**left**) and 1/*T*_2_ (**right**) at 9.4 T of Feridex at various concentrations.

**Figure 2:**
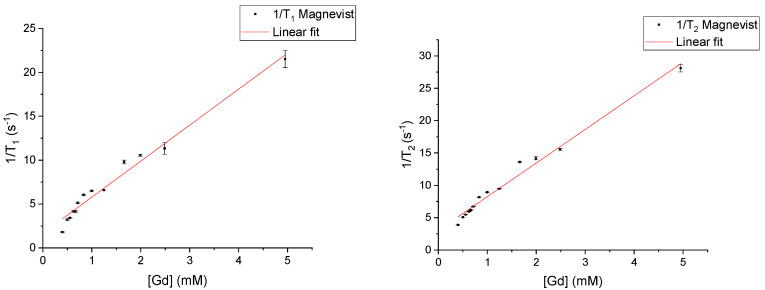
Relaxivity plots showing measured 1/*T*_1_ (**left**) and 1/*T*_2_ (**right**) at 9.4 T of Magnevist at various concentrations.

*T*_1_ and *T*_2_ of the chosen CAs were first measured individually at 9.4 T. Plotted in Figure 1 are the 1/*T*_1_ and 1/*T*_2_ values of various concentrations of Feridex. The highest 1/*T*_2_ values (in the samples with concentrations above 1 mM) had a large uncertainty due to a very short T_2_ relaxation time. The minimum echo time (TE) allowed by the MRI system (10 ms) was too long for the precise measurement of the very short T2 values. Therefore, these results were not included in the figure and subsequent linear fit. In Figure 2, 1/*T*_1_ and 1/*T*_2_ values are plotted for a concentration range of Magnevist. Here, *T*_1_ and *T*_2_ values were measured at 10 mM as well, but these were similarly not included in the figures and linear fits due to their large uncertainties as well as being out of range of what is typically used in the clinic. Using Equations (1) and (2), the relaxivities *r*_1_ and *r*_2_ could be determined by fitting to the measured 1/*T*_1_ and 1/*T*_2_ values of the samples in the dilution series, in a similar fashion to what was performed in previous work [11,12,13]. The slope of a linear fit to the experimental data results in the respective *r*_1_ and *r*_2_ values. The *r*_1_ and *r*_2_ values for Feridex were 2.10 ± 0.13 and 238.97 ± 8.41 mM^−1^s^−1^, respectively. For Magnevist, the respective *r*_1_ and *r*_2_ values were 4.10 ± 0.19 and 5.20 ± 0.20 mM^−1^s^−1^.

The *r*_1_ and *r*_2_ values correspond reasonably well to literature values. The *r*_2_ of Feridex was reported as 307 mM^−1^s^−1^ at 9.4 T [27] and the *r*_1_ of Magnevist as 3.2 mM^−1^s^−1^ at 9.4 T [28]. The *r*_1_ value of Feridex and *r*_2_ value of Magnevist were found for 4.7 T, at 2.3 and 4.0 mM^−1^s^−1^, respectively [25].

The *r*_2_/*r*_1_ ratios were calculated to be *r*_2_/*r*_1_ = 113.8 ± 8.3 for Feridex and *r*_2_/*r*_1_ = 1.27 ± 0.07 for Magnevist. The ratio of Feridex is 89.7 times higher than that of Magnevist. This relative difference in *r*_2_/*r*_1_ ratios compares favorably to the difference demonstrated in the dual contrast work of Anderson et al. [19], who measured *r*_2_/*r*_1_ = 374 for Dy-DOTA-azide and *r*_2_/*r*_1_ = 6.59 for Gd-BOPTA, the relative difference they report being only 56.8 times higher for the Dy-DOTA-azide. Besides the clinical relevancy, the choice of Magnevist and Feridex seems to result in a larger difference of *r*_2_/*r*_1_ values. The larger relative difference would mean that the linear model should be more reliable, due to a smaller chance of overlap in *r*_2_/*r*_1_ errors of the CAs. However, all the CAs discussed have *r*_2_ values higher than *r*_1_, and even the Magnevist we measured is not a “pure” T_1_ contrast agent. These observations indicate that even the combination of a high *T*_1_ and a high *T*_2_ CA cannot be used as a pair of two pure colors that have no overlap in contrast.

The 1/*T*_1_(0) and 1/*T*_2_(0) values were initially determined from the intercept of the linear fit with the y-axes of the data shown in Figure 1 and Figure 2, resulting in values of 1/*T*_1_(0): 0.67 ± 0.17 s^−1^ and 1/*T*_2_(0): 19.47 ± 4.14 s^−1^ for Feridex. 1/*T*_1_(0): 1.69 ± 0.34 s^−1^ and 1/*T*_2_(0): 3.05 ± 0.16 s^−1^ were found for Magnevist. These values appear to vary strongly between contrast agents. A possible reason for the variation in *T*_1_(0) and *T*_2_(0) is the effect of the CA solute on the solubility of O_2_ and CO_2_ in the water, making the background signal not fully independent of the *T*_1_ and *T*_2_ measured from the CA. Averaging the values between CAs resulted in 1/*T*_1_(0): 1.18 ± 0.19 s^−1^ and 1/*T*_2_(0): 11.26 ± 2.08 s^−1^.

As an alternative to the average of the extrapolated y-intercepts, the relaxivity of pure water could be taken as well, to use as 1/*T*_1_(0) and 1/*T*_2_(0) in Equations (3) and (4). The *T*_1_ and *T*_2_ of distilled water were measured, and 1/*T*_1_ was determined to be 0.3687 ± 0.0004 s^−1^ and 1/*T*_2_ to be 2.3474 ± 0.0110 s^−1^. The relaxation rates of pure water are expectedly very low, due to the lack of any magnetic solutes [8]. By comparison, the 1/*T*_1_(0) and 1/*T*_2_(0) values calculated from y-intercepts of the pure CA plots are higher than expected for pure water and show variance between those determined for Feridex and for Magnevist, especially in 1/*T*_2_, resulting in a larger relative error. The results using the 1/*T*_1_(0) and 1/*T*_2_(0) determined from pure water have a smaller relative error and showed a closer similarity overall, resulting in a more conservative model for relaxivities based on CA concentration. Qualitatively, the results between the different 1/*T*_1_(0) and 1/*T*_2_(0) are similar, and our conclusions in this study remained the same between the two approaches. For the demonstration in this work, the pure water relaxivities were used for 1/*T*_1_(0) and 1/*T*_2_(0), as described further on.

### 3.2. Relaxivities of Mixtures of CAs

A series of mixtures of Feridex and Magnevist were prepared, and their *T*_1_ and *T*_2_ were measured. Using the proposed Equations (3) and (4) and thus assuming completely linear behavior, analytically predicted *T*_1_ and *T*_2_ values of the mixtures were calculated. For 1/*T*_1_(0) and 1/*T*_2_(0), the relaxivities of distilled water were used, resulting in 1/*T*_1_(0) = 0.3687 ± 0.0004 s^−1^ and 1/*T*_2_(0) = 2.3474 ± 0.0110 s^−1^.

The calculated results were compared in three-dimensional plots to the experimentally determined *T*_1_ and *T*_2_ of mixtures at corresponding concentrations shown in Supplementary Materials (Figures S1 and S2). In these figures, it can be seen that the calculated values do not fully overlap with the measured ones. In particular, the higher concentrations of Feridex in the *T*_2_ measurements correspond with a large deviation from the predicted values.

**Figure 3:**
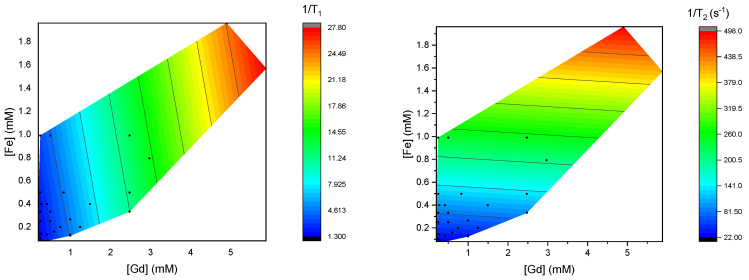
Contour color plots of calculated 1/*T*_1_ values (**left**) and 1/*T*_2_ values (**right**) of the sampled mixtures of Feridex and Magnevist. Equations (3) and (4) were used, together with 1/*T*_12(_0) values of pure water.

**Figure 4:**
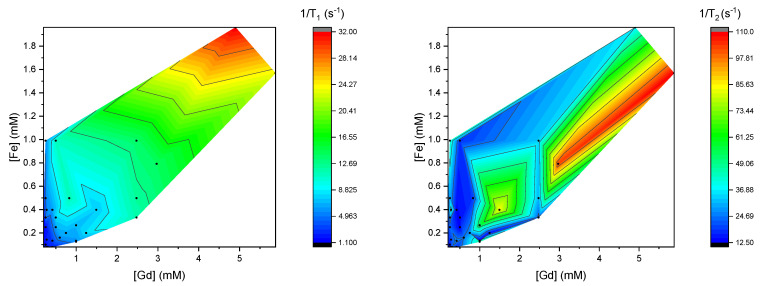
Visualization of measured 1/*T*_1_ (**left**) and 1/*T*_2_ (**right**) at 9.4 T of mixtures of various concentrations of Magnevist and Feridex in contour color plots.

To qualitatively compare the trends in the relaxivity data, contour color plots were generated of the calculated 1/*T*_1_ and 1/*T*_2_ values, in Figure 3, and of the measured 1/*T*_1_ and 1/*T*_2_ values, in Figure 4. In these plots, blue represents a low 1/*T*_1_ or 1/*T*_2_, and red represents the high value, as indicated in the color bar. The black lines correspond with the tick marks on the color bar.

In the calculated 1/*T*_1_, a gradual rise in relaxivity is observed, that seems to scale with both the concentration [Gd] and [Fe], as predicted from the positive *r*_1_ and *r*_2_ values of these contrast agents. As expected from the calculated r_1_ values, it scales more with the concentration of Magnevist. For 1/*T*_2_, a stronger dependence on the concentration of Feridex is observed, as a result of its higher *r*_2_.

When the trends in the calculated relaxivities are compared qualitatively to those measured, a few differences are observed. In the measured 1/*T*_1_ plot, 1/*T*_1_ appears to depend more strongly on the concentration [Fe] than in the calculated plot. In the measured 1/*T*_2_ plot, the results seem more irregular for the different concentrations. Compared to the calculated values, the 1/*T*_2_ seems to scale a lot less strongly with the concentration [Fe].

**Figure 5:**
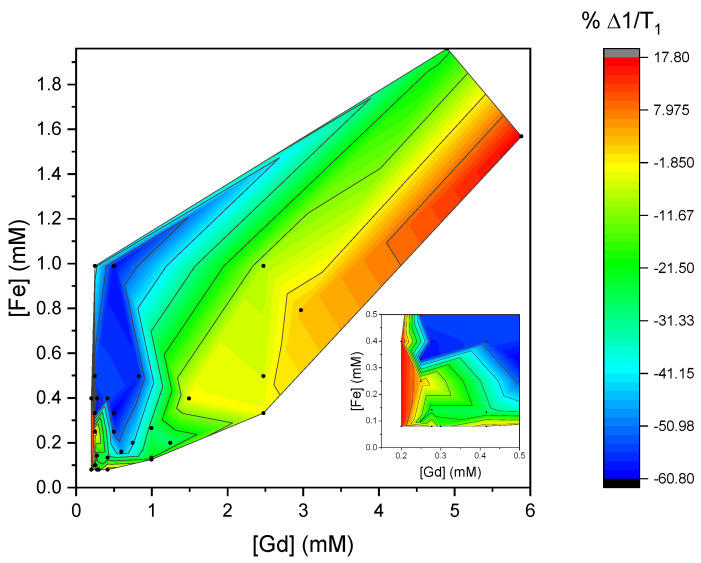
Color map plot of the difference between the calculated 1/*T*_1_ (using 1/*T*_1_(0) from water) from the linear addition of *r*_1_ values determined from pure mixtures and the measured 1/*T*_1_ at the same concentration. The percent difference of the calculated values with respect to measured values is shown. The insert is a zoom-in of the region at low concentrations. In this graph, the yellow-colored region shows the smallest deviation from linearly calculated values.

**Figure 6:**
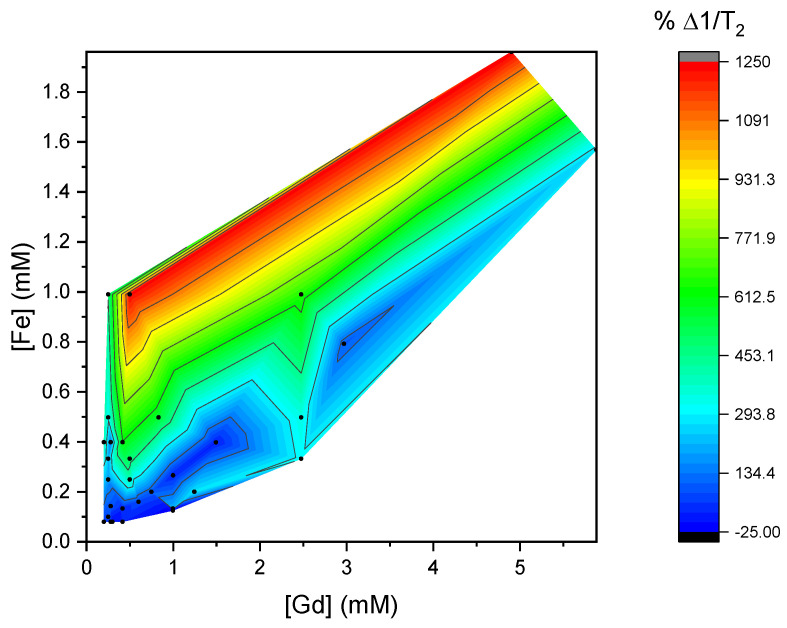
Color map plot of the difference between the calculated 1/*T*_2_ (using 1/*T*_2_(0) from water) from the linear addition of *r*_2_ values determined from pure mixtures and the measured 1/*T*_2_ at the same concentration. The percent difference of the calculated values with respect to the measured values is shown. In this graph, the blue-colored region shows the smallest deviation from linearly calculated values.

To visualize specifically the differences between experimental and calculated results, contoured color maps of the deviations were generated. The differences Δ1/*T*_1_ and Δ1/*T*_2_ were calculated by subtracting the calculated relaxivity times from those measured at the same concentration. Figure 5 shows a color contour map of the difference Δ1/*T*_1_ at various mixture concentrations, and Figure 6 shows a color contour map with the Δ1/*T*_2_ at these concentrations.

The calculated values are observed to deviate from the measured 1/*T*_1_ values in the case of low concentrations of Gd^3+^. In the low extreme, 1/*T*_1_ deviates by around −60% at the combination of concentrations [Gd] of 0.2 mM–1 mM and concentrations [Fe] of 0.2 mM–1 mM. The deviations from the calculated values appear to be the highest at the lower concentrations of Gd^3+^ and start to correspond more closely to the predicted value as the concentration increases.

A physical explanation for the underestimation of 1/*T*_1_ from the linear model lies in the influence of the magnetization of the Feridex on the *r*_1_ of Magnevist. The Gd^3+^ ions in Magnevist have a relatively large r_1_ (4.10 mM^−1^s^−1^), which is dependent on its magnetic moment and electronic relaxation time *T*_1e_. Interaction with the strongly magnetizable iron oxide nanoparticles may lead to an increase in the Gd^3+^ magnetic moment, and thus a larger effective *r*_1_. The observed synergistic enhancement in relaxivity when combining ions, such as Gd^3+^ and Fe^3+^ in aqueous solutions, have been explained by several interrelated mechanisms, including dipolar coupling, magnetic interactions, and water exchange rate modulation [8,29,30]. This synergistic effect between CAs has been observed in NaGdF_4_-coated NaDyF_4_ NPs, where the highly paramagnetic Dy^3+^-based core enhanced the *r*_1_ of Gd^3+^ ions in the shell [31]. This simultaneous dependence of the r_1_ of a single contrast agent on the concentration of both contrast agents forms a complication for the use of the linear model in a clinic, where it is not known a priori how the contrast agents will be distributed at the location of interest.

In the measured 1/*T*_2_ values, a much larger deviation from the corresponding calculated values can be seen. The lowest deviation is −25% at concentrations of about 0.1 mM [Fe]. At higher concentrations than this, the deviation Δ1/*T*_2_ increases gradually further, reaching extreme differences of 1250% at the highest concentrations of Feridex, where [Fe] is around 1.9 mM. At concentrations higher than ~0.2 mM [Fe], the *T*_2_ values do not scale linearly with the concentration of the contrast agents.

We speculate that this overestimation of 1/*T*_2_ from the linear model is due to the complex interaction between ions, including the magnetic field perturbations generated by the superparamagnetic nanoparticles that enhance the spin–spin relaxation of protons. The interactions may become higher at higher concentrations, meaning that the number of protons affected by the magnetic field does not increase linearly with the number of nanoparticles in dispersion. To further verify this effect, it would be interesting to measure 1/*T*_2_ values at high concentrations of *T*_2_ contrast agents. Using our MRI analysis, the errors in *T*_2_ at high concentrations of pure Feridex became too large for reliable analysis.

From the comparisons between calculated and experimental 1/*T*_1_ and 1/*T*_2_ data, it can be concluded that there is only a small range of concentrations in which the relaxivities are linearly additive. This is impractical for clinical use, where concentration can widely vary with the amount of contrast agent that reaches the area of interest (e.g., the tumor). These results are highly important for the proposed application of correlation MRI, where the concentration of *T*_1_- and *T*_2_-specific contrast agents could potentially be very low, or very high. It was calculated that, from a typical (0.25 mL) intravenous nanoparticle injection into mice developed with a LNCaP tumors before MRI, the concentration at the tumor location can typically be between 1 and 4 mM depending on tumor size, assuming a 1.5% localization of the injected contrast agent inside the tumor [31]. This suggests that the concentration range measured is clinically relevant both in the low and the high end. Especially since CAs that specifically target locations of interest are in development, higher concentrations of localized CA become increasingly clinically relevant.

We investigated the possibility of overcoming the non-linear behavior of the *T*_1_ and *T*_2_ contrast agent mixtures by using the three-dimensional data of the mixtures as a calibration curve. These results are discussed in Supplementary Materials. Polynomial curves were fitted to the 1/*T*_1_ and 1/*T*_2_ data of the series of mixtures (Figure S3). The polynomials were then used to demonstrate the use of these curves by approximating the concentration of a sample based on its 1/*T*_1_ and 1/*T*_2_ value (Figure S4). In this specific demonstration, the predicted [Gd] was 32% lower than the real concentration, and the [Fe] was 18% higher. While a demonstration of concentration determination based on the measured relaxivity data of the mixtures could be shown, the accuracy of the model should be improved by including more samples in the experimental data to determine the calibration curve.

### 3.3. Theoretical Insight

The fundamental reason for the deviations in the relaxation rates 1/*T*_1_ and 1/*T*_2_ is the strong magnetic dipole–dipole interactions between the electron spins of the CA molecules. To understand this, one should start with a single CA and low concentrations. Actually, “low concentration” is meant as a concentration range in which the effective (averaged) distance between the paramagnetic molecules is sufficiently large enough to make the effects of their mutual interactions negligible. The ^1^H relaxation results from the proton–electron dipole–dipole coupling which fluctuates in time as a result of molecular motion (translational diffusion of water molecules (outer-sphere relaxation), tumbling of the paramagnetic species with bound water molecules (inner-sphere relaxation) mediated by water exchange processes, and electron spin relaxation. In other words, electron spin relaxation acts as an additional source of modulation of the proton–electron dipole–dipole coupling. Electron spin relaxation is in most cases caused by Zero Field Splitting (ZFS) interactions modulated by internal dynamics of the paramagnetic complex. This represents a hierarchy of events: the electron spin relaxation is independent of the presence of the ^1^H nuclei (ZFS is a one-spin interaction involving only the electron spin), but the ^1^H relaxation is strongly influenced by the electron relaxation. The simplest model of paramagnetic relaxation enhancement (PRE) includes only two electronic relaxation times (the spin–lattice and the spin–spin) and accounts for their frequency (magnetic field) dependencies [32,33]. More elaborated (and realistic) theoretical models take into account several electronic relaxation rates (and their frequency dependencies) associated with different spin coherences (one should remember that the electronic spin quantum numbers are high, for instance 7/2 for gadolinium, and this implies a set of electronic relaxation rates) [34,35,36,37]. The interplay between the electronic relaxation times (that depend on the resonance frequency) and the dynamical parameters (rotational correlation time, translation diffusion coefficient, and exchange life time) creates a set of correlation times that modulate the proton–electron dipole–dipole coupling. Depending on the resonance frequencies, changes in the correlation times lead to different proton relaxation effects.

The picture becomes even more complicated when the electronic relaxation includes a second contribution—the one caused by electron–electron spin and dipole–dipole couplings. Moreover, these interactions are modulated by the relative translation diffusion of the interacting species. The presence of the additional relaxation channel for the electron spin leads to an effect where a further increase in the concentration of the paramagnetic molecules does not imply a proportional enhancement of the ^1^H relaxation (the increase in the relaxation rates is much lower, if any) because the electron spin relaxation becomes faster. Some deviations from the linearity are already observed in Figure 1 and Figure 2. The physical mechanism is analogous when two kinds of CAs are present; however, in such cases, relationships between r_1_ and r_2_ for both CAs introduce further complications by enhancing or reducing the influence of the electronic relaxation rates associated with specific spin coherences of the ^1^H relaxation.

Although we are not of the opinion that efforts should not be made to model these processes in details, phenomenological approaches are of great value for both the application of and fundamental science purposes.

## 4. Conclusions

The viability of using a linear model to quantify two contrast agents simultaneously for a *T*_1_ and *T*_2_ MRI correlation technique at 9.4T was verified. *T*_1_ and *T*_2_ were measured from a series of commercially available Magnevist (Gd^3+^ multidentate complex) and Feridex (iron oxide nanoparticles) dispersions. From these, specific relaxivities were calculated to be *r*_1_ = 2.10 ± 0.13 and *r*_2_ = 238.97 ± 8.41 mM^−1^s^−1^ for Feridex and *r*_1_ = 4.10 ± 0.19 and *r*_2_ = 5.20 ± 0.20 mM^−1^s^−1^ for Magnevist. The values were used to predict *r*_1_ and *r*_2_ values of mixtures of the two contrast agents. In 3D curves, the calculated values were compared to values measured at the same concentrations. It was found that the 3D curves correlated closely for *T*_1_ values at concentrations above 1.5 mM [Gd]. Below these concentrations, the measured values started to deviate from those calculated. For *T*_2_ values, a difference was observed at higher concentrations of Feridex. Especially above 0.2 mM [Fe], the experimental data started to deviate strongly from the calculated data. It was suggested that the 1/*T*_2_ does not scale linearly with the concentration of the *T*_2_-active contrast agents. To overcome this problem in correlation MRI, it was proposed that 3D curves of the relaxation times of mixtures should be used, and a demonstration of the concentration determination using polynomial fits of the measured relaxivity data from the mixtures was performed, but the accuracy should be improved by having a larger amount of samples in the experimental data.

One of the findings of the experimental results was also observations that, at high concentrations of Feridex in the mixture, the 1/T_2_ values were much lower than expected from “pure” Feridex. We anticipate that this phenomena is caused by Magnevist reducing the colloidal stability of Feridex. The aggregation causes much faster relaxation than “free” NPs.

The proposed 3D calibration model, which accounts for the field strength dependence of relaxivity, may remain valid at clinical 3T or 1.5T. However, the accuracy of the model could be influenced by the different relaxivity ratios (r_2_/r_1_) at these field strengths. At lower field strengths, the r_2_/r_1_ ratio tends to increase: for Feridex, r_2_/r_1_ is ~150 and ~1.2 for Magnevist at both 1.5T and 3T, while at 9.4T r2/r1 is ~114 for Feridex and 1.3 for Magnevist [this report and [38]]. Although differences in r2/r1 ratio are not large, they could affect the model’s performance.

While the demonstration of the concept was provided in vitro, future work should involve measurements on tissue-mimicking phantoms, or in vivo. Although the general concept would likely remain, the in vivo experiments could provide altered results due to the different anatomy and physiology of tissues, including viscosity, vasculature, protein content, etc. Our results further highlight the need for a more elaborate physical model to describe the effect of the interaction between different CAs on the overall *T*_1_ and *T*_2_. For example, the studies of stability of the agents at higher concentrations with Dynamic Light Scattering (DLS) could provide an explanation for the rather unexpected shortening of the relaxation times. Application of a dedicated NMR spectrometer would deliver more precise measurements of the relaxation times, particularly at high concentrations. These and other instruments would likely provide definite and broader explanation of the results, including possible multi-exponential behavior of the relaxation curves. The deviations from the linear model fall in the clinically relevant concentration range, and mean that for the practical use of correlation MRI, two contrast agents cannot be quantified using this linear addition model.

## Data Availability

The original contributions presented in this study are included in the article/Supplementary Materials. Further inquiries can be directed to the corresponding authors.

## Supplementary Materials

The following supporting information can be downloaded at: https://www.mdpi.com/article/10.3390/ma18102290/s1, Figure S1: 3D plot of measured 1/*T*_1_ at various concentrations of Feridex and Magnevist mixtures; Figure S2: 3D plot of measured 1/*T*_2_ at various concentrations of Feridex and Magnevist mixtures; Figure S3: polynomial calibration fits; Figure S4: calibration-based concentration estimation for a blind sample.
