# Supplementary material for: Utilizing T1- and T2-Specific Contrast Agents as “Two Colors” MRI Correlation
Source: Materials (Basel). 2025 May 14;18(10):2290. doi: 10.3390/ma18102290 (PMC12113452; doi:10.3390/ma18102290)
Supplement: Supplementary file 1 [file materials-18-02290-s001.zip › materials-3595075-supplementary.pdf]

Supporting information to

Utilizing T_1- and T_2- Specific Contrast Agents as "Two Colors" in MRI Correlation

Adriaan L. Frencken*[1,2], Barbara Blasiak*[3,4], Boguslaw Tomanek[3,4], Danuta Kruk[5], Frank, C. J. M. van Veggel[1,2]

[1]*Department of Chemistry, University of Victoria, Victoria, British Columbia, V8W 2Y2, Canada*
[2]*Centre for Advanced Materials & Related Technologies (CAMTEC), University of Victoria, Victoria, British Columbia, V8W 2Y2, Canada*
[3]*Experimental Imaging Centre, University of Calgary, Calgary, Alberta T2N 4N1, Canada*
[4]*Institute of Nuclear Physics, Polish Academy of Sciences, Krakow, 31-342, Poland*
[5]*Department of Mathematics and Computer Science, University of Warmia and Mazury, Olsztyn, 10-710, Poland*

I. 3-Dimensional plots

The calculated results were compared to the experimentally determined T_1 and T_2 of mixtures at corresponding concentrations in Figures A1 and A2. In these figures, it can be seen that the calculated values do not fully overlap with the measured ones. In particular, the higher concentrations of Feridex in the T_2 measurements correspond with a large deviation from the predicted values.

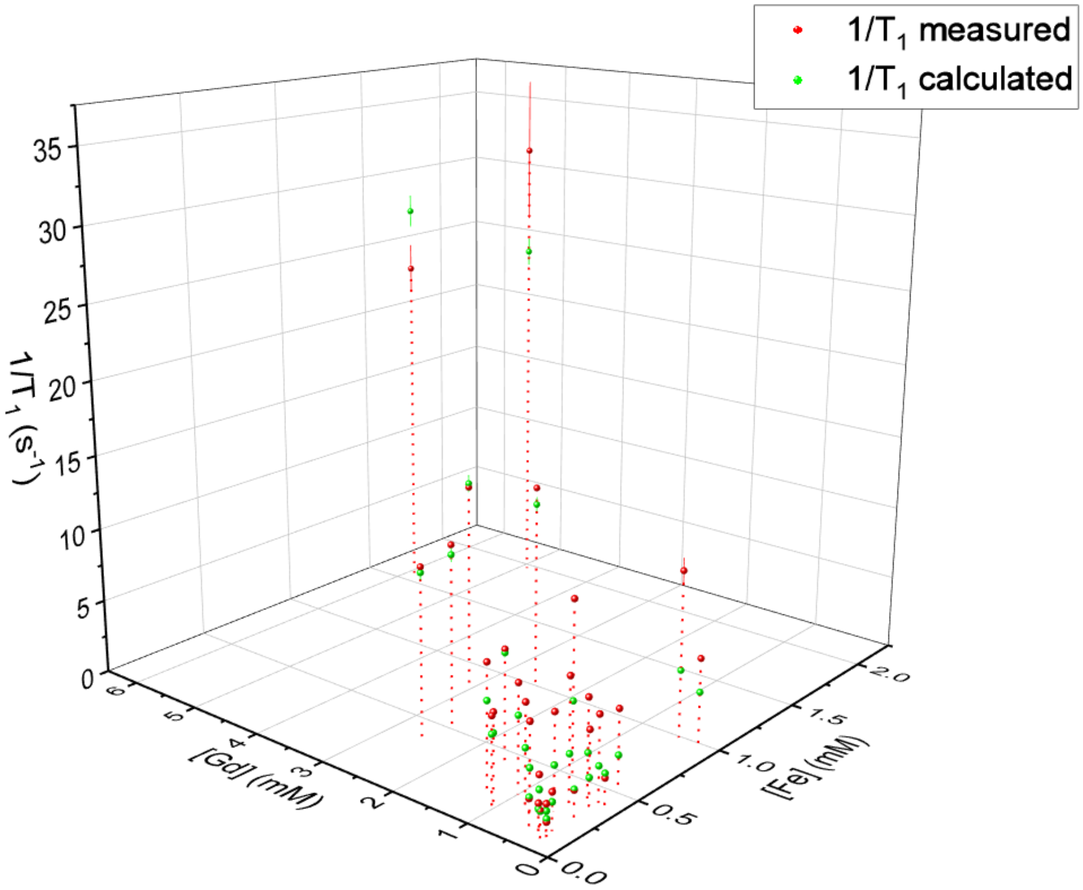

Figure S1: 3D plot of the measured 1/T_1 at various concentrations of Feridex and Magnevist mixtures.

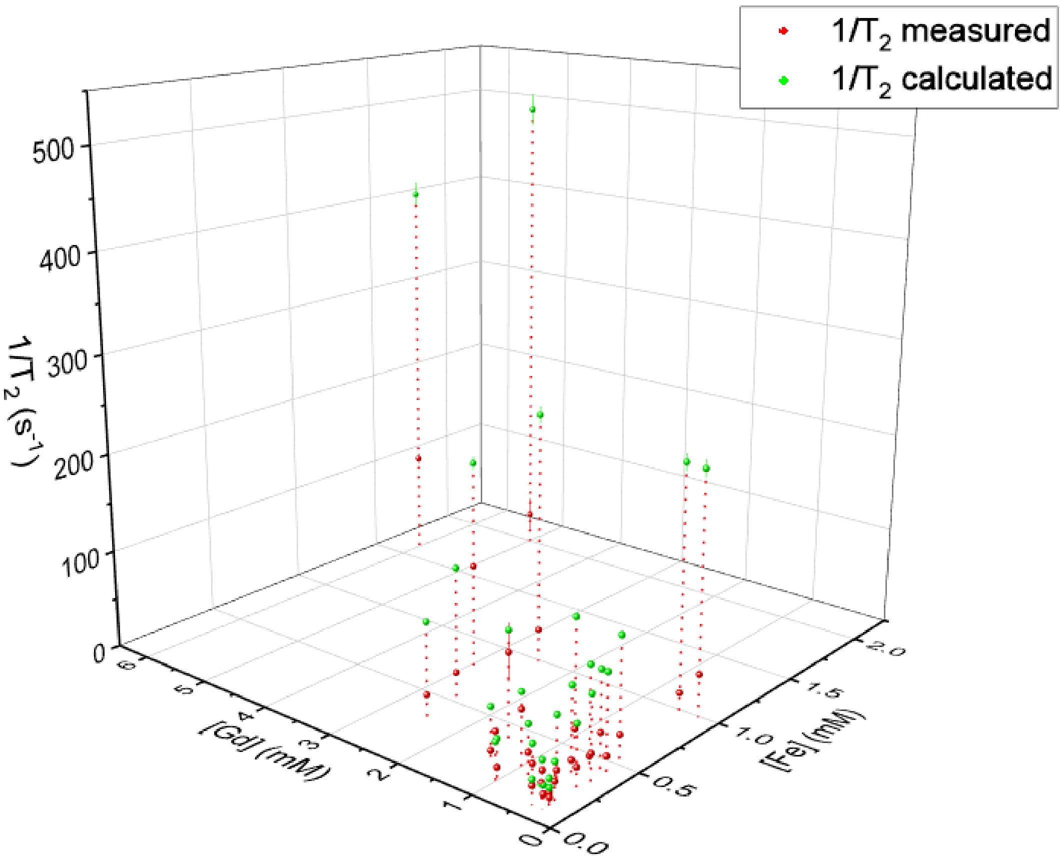

Figure S2: 3D plot of the measured $1/T_2$ at various concentrations of Feridex and Magnevist mixtures.

II. Calibration curve

It is proposed that to utilize a specific T_1 contrast agent in conjunction with a specific T_2 contrast, a 3-dimensional calibration curve needs be generated to predict the concentrations from $1/T_1$ and $1/T_2$ values outside the linear region. As an approach to generate a 3D calibration curve, polynomials (of the form shown in equation (A1)) were fit to the plot of concentrations [Fe] and [Gd] vs. $1/T_1$, and to the plot of those concentrations vs. $1/T_2$. The results of these fits are shown in Figure A3. The fit through the $1/T_1$ data is reasonable, with an R^2 of 0.945, whereas the fit through $1/T_2$ is less good with R^2 = 0.557. The poor fit in $1/T_2$ can be attributed to the larger variation [10,11]in measured $1/T_2$ values, as shown in the main text in Figure 6 (right). It can be seen that outside the range of the measured samples, curves show drops down towards lower relaxivities, notably at high [Gd] in $1/T_1$ and high [Fe] in $1/T_2$. A drop in relaxivity with the increased concentration is not expected, and this is assumed to be a result of under-sampling in this range of the experimental data to which the polynomial is fitted.

$$z = p_{00} + p_{10} * x + p_{01} * y + p_{20} * x^2 + p_{11} * x * y + p_{02} * y^2 \quad \text{(A1)}$$

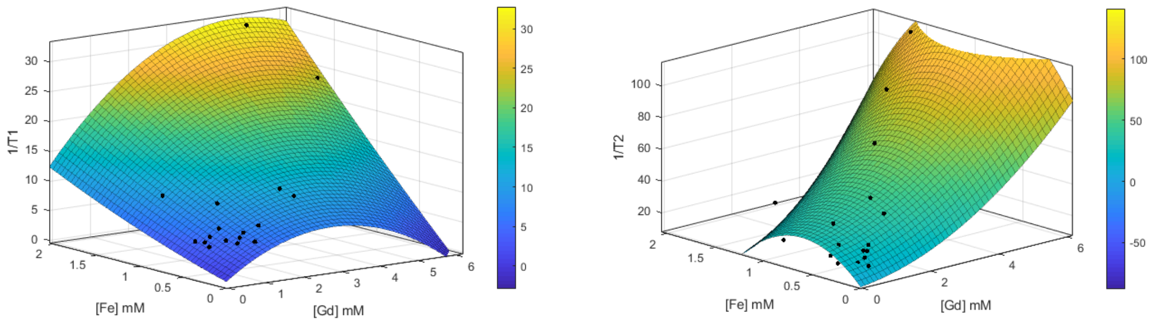

Figure S3: Measured $1/T_1$ values (left) and $1/T_2$ values (right) of mixtures (black dots), with corresponding fits to polynomial functions.

An example demonstration of the potential use of these calibration curves to determine the concentrations in a mixture of Feridex and Magnevist of unknown composition, based on just the measured $1/T_1$ and $1/T_2$ values, is shown in Figure A4. Here, contour color plots are shown for the generated fits, and a red line shows the specific $1/T_1$ measured in the unknown sample, a green line shows the specific $1/T_2$. From readout of the crossing point of these lines, corresponding [Gd] and [Fe] can be determined. For the unknown sample, the $1/T_1$ = 10.86 s^{-1}, and $1/T_2$ = 17.95 s^{-1}. [Gd]= 0.567 mM, and [Fe] = 1.173 mM were determined from the crossing point. The actual concentrations were [Gd] = 0.831 mM,

and [Fe] = 0.990 mM. There is still a difference between the real and predicted concentration, the predicted [Gd] was 32 % lower than the real concentration, and the [Fe] was 18 % higher.

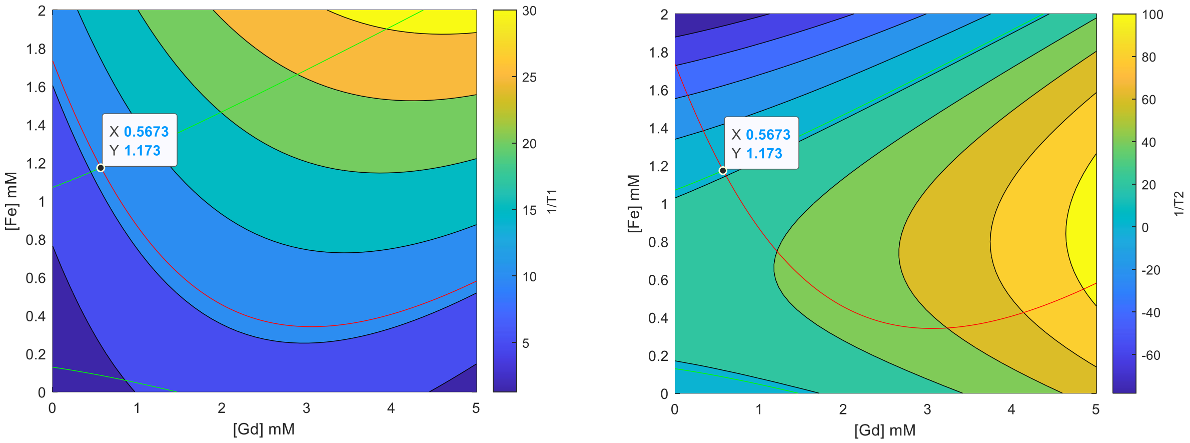

Figure S4: Demonstration of calibration curves based on polynomial fits to experimental data. An example determination is shown of a blind sample with $1/T_1$ = 10.86 s^{-1}, $1/T_2$ = 17.95 s^{-1}. From the crossing points, [Gd]= 0.567 mM, and [Fe] = 1.173 mM were determined. The actual concentrations were [Gd] = 0.831 mM, and [Fe] = 0.990 mM.

While a demonstration of the calibration curve could be given in certain ranges, it is not yet in its optimal form. Notably, no measured sample data in the range of low [Fe] and high [Gd], and *vice versa*, was available for the generation of the polynomial fits. In these concentration regions, it was not possible to determine the concentration of Feridex or Magnevist based on the relaxivity of a mixture. To overcome this problem, samples need to be measured in the entire range of possible concentrations relevant for clinical use. Furthermore, it was noted that a high degree of irregularity existed for mixtures in the measured $1/T_2$. This has resulted in a fit with a low R^2, and is expected to yield a high error in quantitative determination of concentrations. This can also be addressed by sampling a larger number of samples, potentially increasing the accuracy of $1/T_2$ determination in the concentration ranges of interest.

It should also be noted that the effective r_1 and r_2 of CAs inside living tissue may deviate from those in pure water, due to differences in the chemical and magnetic environment. These deviations need to be taken into consideration for a quantitative model as well.
